# Enhancing Precision: A Visual Guide to Drill-Assisted External Dacryocystorhinostomy Surgery

**DOI:** 10.7759/cureus.54110

**Published:** 2024-02-13

**Authors:** Joobin Khadamy

**Affiliations:** 1 Ophthalmology, University Hospital of Umeå, Umeå, SWE; 2 Ophthalmology, Skellefteå Eye Clinic, Skellefteå, SWE

**Keywords:** microperforator or mechanical burr or electric drill assisted external dacryocystorhinostomy (dcr), dcr video, silicone intubation during dcr, high-speed drill, nldo, nasolacrimal duct obstruction, microperforator assisted external dacryocystorhinostomy (dcr), micro drill assisted external dacryocystorhinostomy (dcr), micro-perforation, external dacryocystorhinostomy

## Abstract

This technical report aims to provide a visual guide to the drill-assisted external dacryocystorhinostomy (DCR) technique with silicone intubation. Through a step-by-step video demonstration, it addresses inherent documentation challenges and highlights crucial considerations. A critical aspect of the procedure's success lies in creating a clear space around the drilling area to prevent thermal burns and soft tissue wrapping around the burr. Additionally, it emphasizes the careful use of smaller burr diameters and the importance of drilling techniques, advocating for minimal perpendicular drilling while maintaining rotational polishing movements to minimize the risk of rapid penetration and potential nasal mucosal injury.

The thermal cauterization of perforating nutrient vessels during bone drilling offers a significant advantage in reducing the risk of bleeding. A review of existing limited studies comparing drill-assisted and conventional external DCR reveals advantages such as shorter surgical duration, lower intraoperative hemorrhage rate, more regular osteotomy edges, increased ostomy patency, and potential prevention of soft tissue or mucosal injuries. Nonetheless, achieving these benefits necessitates enhanced hand and foot coordination. However, despite these benefits, a noticeable gap exists in the literature concerning comprehensive studies and comparative analyses. Furthermore, exploring the associated cost and learning curve of adopting this surgical technique is essential.

This report aims to fill the existing gap in the literature and serve as a visual reference for surgeons interested in adopting drill-assisted external DCR.

## Introduction

Common indications for dacryocystorhinostomy (DCR) include primary acquired nasolacrimal duct obstruction (NLDO), secondary acquired NLDO resulting from factors such as prior midfacial trauma, chronic nasal or sinus inflammation, nasal surgery, neoplasms, or dacryoliths, functional obstruction of outflow due to lacrimal pump weakness or facial nerve palsy, congenital NLDO after failed prior probing or intubation, acute dacryocystitis unresponsive to medical treatment, and chronic dacryocystitis. In the ever-growing landscape of endoscopic techniques driven by aesthetic concerns, external DCR remains the gold standard for addressing NLDO [1-3].

A pivotal element in ensuring the success of external DCR lies in the precise creation of the ostomy. The conventional mechanical osteotomy, often executed with Kerrison Rongeur or bone punches, poses challenges in cases with thick bones and requires blind fracture induction. This method carries the inherent risk of nasal mucosal damage and subsequent bleeding, potentially compromising flap creation, intraoperative visualization, and overall procedure success [1-3].

In contrast, high-speed surgical drills present an alternative approach, eliminating the need for bone fracture induction and reducing the risk of mucosal damage. Another advantage lies in the thermal cauterization of perforating vessels during bone drilling, minimizing the risk of bleeding.

Despite the clinical significance of this technique, the visualization of surgical steps is challenging due to the limited field of operation, and the current literature lacks comprehensive video guidance for drill-assisted external DCR [3-7]. This technical report aims to fill this gap by providing a visual guide to the drill-assisted external DCR technique with silicone intubation. Essential surgical considerations are highlighted to optimize outcomes and minimize complications in drill-assisted external DCR.

## Technical report

Drill-assisted external DCR is a routine procedure at our center, facilitated by our shared operation rooms with the Otorhinolaryngology (ENT) department. The accessibility of drills and the exchange of experiences with our ENT colleagues have historically aided in integrating this technique into our standard practice.

This report serves as a visual guide and demonstrates the steps and essential considerations leading to a drill-assisted external DCR. Video 1 highlights technical steps. The video compilation aims to offer the optimal visual representation of the surgical technique by combining insights from various cases. This approach allows for a more detailed exploration of each step.

**Video 1:** Step-by-step guide to drill-assisted external DCR surgery The video is mixed to offer the optimal visual guide, seamlessly integrating each step. For optimal viewing experience, we recommend watching this video on a mobile device. This will allow you to fully appreciate the detailed visual demonstration of the drill-assisted external dacryocystorhinostomy (DCR) technique with silicone intubation. By viewing on a mobile device, you can easily navigate through the steps and gain a comprehensive understanding of the procedure. DCR: Dacryocystorhinostomy

**Figure 1:**
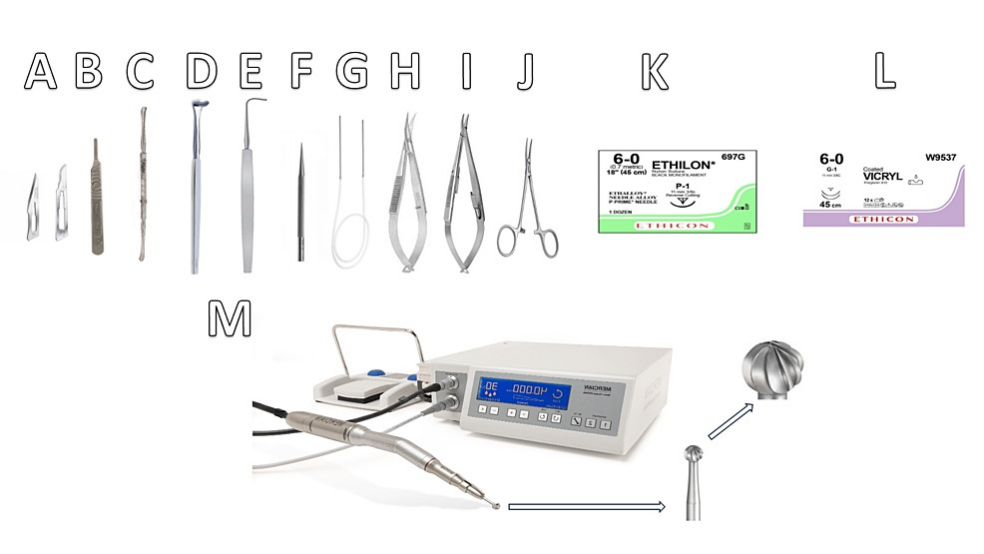
Essential instruments for drill-assisted external DCR A: Straight number 11 ( Left) and curved number 15 blade (Right). B: Scalpel handle. C: Freer's periosteal elevator. D: Desmarres retractor 8-12 mm. E: Graefe muscle hook. F: Punctal dilator. G: Bicanalicular stents. H: Westcott Scissor. I: Castroviejo curved needle holder. J: Curved mosquito forceps. K: Nylon 6-0 curved suture. L: Vicryl 6-0 curved suture. M: High-speed surgical drill with round cutting 5-8 mm burr. DCR: Dacryocystorhinostomy

Moreover, the depiction of essential instruments enhances the clarity and accessibility of the presented information (Figure 1). In addition to these instruments, it is imperative to have a Fraizer suction tube and cautery readily available. Silk 4-0 sutures are also indispensable for achieving hemostasis or providing traction during the procedure.

Preoperative assessments

Preoperative assessments forPreoperative assessments for DCR involve gathering a detailed medical history, conducting a physical examination of the nasal and periorbital regions, and performing diagnostic tests such as imaging studies to evaluate the nasal anatomy and tear drainage system. Ocular assessments, including tests for tear production and drainage function, are also essential, along with evaluating the nasal septum and turbinates for any abnormalities. Coagulation status is assessed to identify potential bleeding risks, and patients are educated about the procedure, expected outcomes, and postoperative care. These assessments enable surgeons to tailor the surgical approach, anticipate challenges, and optimize patient outcomes.

Anesthesia

Anesthesia plays a crucial role in external DCR procedures, with general anesthesia typically preferred, although regional blocks for local anesthesia remain an alternative. Some surgeons recommend an additional injection of local anesthesia, typically a combination of lidocaine (1-2%), bupivacaine (0.5%), and epinephrine (1:100,000), at the incision site to minimize intraoperative bleeding and post-operative discomfort. Further risk reduction measures include maintaining low blood pressure and adopting a reverse Trendelenburg position with head elevation (15⁰). Additionally, placing ribbon gauze or mesh soaked in adrenaline (2 mL of 1:1000) between the inferior turbinate, nasal septum, and passing it superiorly and posteriorly into the middle meatus before surgery (10 minutes) can enhance hemostasis.

**Surgical Gem: **Applying ointment to both eyes at this stage can reduce the risk of exposure keratopathy and potential corneal damage during instrumentation.

Surgical steps

**Figure 2:**
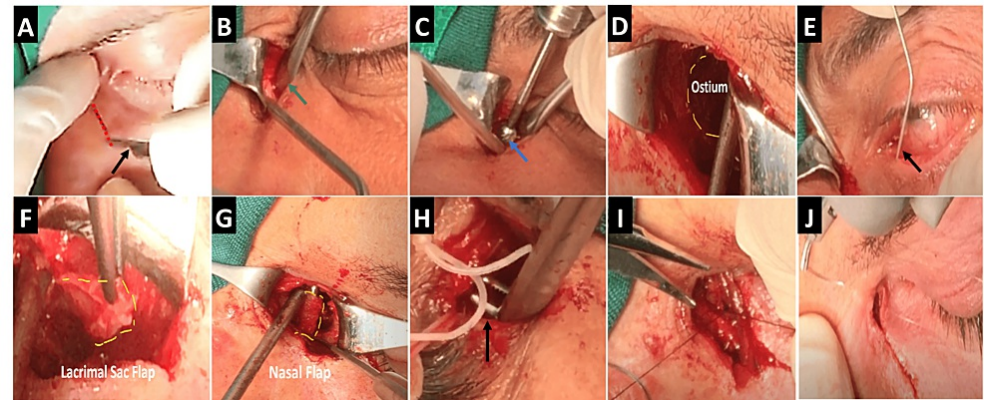
Steps of drill-assisted external DCR surgery A: A linear incision (Red dashed line) is made using a number 15 blade (Black arrow). B: Dissection of orbicularis oculi, periosteum, and lacrimal sac is performed using a Freer periosteal elevator 7.5" (Green arrow). C: Drill-assisted osteotomy is carried out using a 5-8 mm diameter round cutting burr (Blue arrow). Residual bones on the floor of the ostium can be removed with a forceps. D: The result is a round and regular ostium (Yellow dashed semicircle). E: Placement of a bent stainless steel probe (Black arrow) to insert a silicone tube. F: Creation of the lacrimal sac flap (Yellow dashed line). G: Creation of the nasal mucosal flap (Yellow dashed line) using a blade number 11 and muscle hook. H: Passing the silicone tube to the nasal cavity through the ostium with the assistance of a curved mosquito forceps (Black arrow). I: Suturing the flaps together, as well as the orbicularis oculi muscle, in a layered fashion using Vicryl 6-0. J: Skin closure with a continuous subcuticular technique using a nylon 6-0 suture. DCR: Dacryocystorhinostomy

The forthcoming section provides a detailed exploration of the surgical steps involved in drill-assisted external dacryocystorhinostomy (DCR), complemented by a visual overview encapsulated in Figure 2. Each step is intricately presented to enhance clarity and serve as a visual resource for surgeons seeking to adopt this specialized technique.

Step 1: Skin Incision

**Figure 3:**
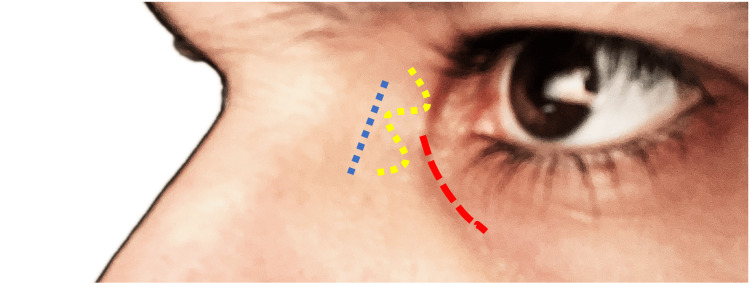
Incision patterns in external DCR This image illustrates different incision patterns utilized in external dacryocystorhinostomy (DCR) procedures. The linear incision (Blue) is made on nasal sidewall, the W-form incision (Yellow) offers an alternative approach, while the curvilinear incision (Red) is typically made along the anterior lacrimal crest. The incision should be lateral to the angular vein but medial or inferior to the medial canthal ligament. DCR: Dacryocystorhinostomy

A 10-20 mm skin incision is created 8-10 mm nasal to the medial canthus using a 15-number blade (Figure 2A). It is crucial to cut through the layers down to the periosteum in a single motion. This approach aids in layer dissection and minimizes the risk of shredding the orbicularis oculi muscle fibers during periosteal elevator use. The incision can adopt various patterns, including linear, curvilinear, or W-form (Figure 3). While literature lacks consensus on the most cosmetically favorable pattern [8], curvilinear incisions are generally associated with a risk of web formation in susceptible patient groups.

Surgical Gem: Incision should be made lateral to the angular vein.**Surgical Gem:**

**Surgical Gem: **If angular vein is injured during surgery, achieving hemostasis involves passing a round silk 4-0 suture through the skin, deeper than the level of the vessel, at both proximal and distal sites relative to the injury site.

Step 2: Dissection of Soft Tissue

The orbicularis oculi, periosteum, and lacrimal sac are dissected using a Freer periosteal elevator (Figure 2B). Care should be taken to preserve the integrity of the medial canthal tendon, dissecting it only when necessary. Proper cauterization not only reduces hemorrhage but also ensures optimal visualization during dissection.

**Surgical Gem: **Proper dissection enhances visualization and reduces the risk of soft tissue burning and entanglement around the burr during drilling.

Step 3: Drill-Assisted Osteotomy

Drill-assisted osteotomy utilizes a 5-8 mm diameter round cutting burr (Figure 2C), although a smaller burr size may offer improved penetration, it also heightens the risk of rapid perforation of the underlying nasal mucosa and potential wrapping around the burr. To ensure precision during the procedure, it is crucial to move the drill rotationally and parallel to the bone plane, akin to polishing movements, while enlarging the ostium. This technique helps maintain accuracy and control, facilitating the creation of a smooth and well-defined ostium essential for optimal surgical outcomes. The polishing movements should be continued until the nasal mucosa becomes visible beneath a thin bony layer or fragment. Any residual bones on the ostium's floor can be extracted using forceps.

**Surgical Gem:** Keep surrounding tissues away to reduce the risk of thermal burn or burr wrapping.

Step 4: Placement of Silicone Tubes in the Canaliculus

Following punctum dilation, probes are passed through the canaliculi (Figure 2E). Less experienced surgeons may find the use of metallic probes helpful in localizing the lacrimal sac.

Step 5: Creation of Flaps

The flaps are created through initial parallel incisions, about 1 cm apart, using a number 11 blade. A muscle hook is then passed through these parallel incisions, and the posterior connection is scissored using a Westcott scissor or number 11 blade (see Video 1). The flaps should have equal width and length for optimal surgical outcomes.

**Surgical Gem:** The nasal flap (Figure 2G) should be created later to avoid potential bleeding that may compromise subsequent stages of the procedure.

Step 6: Passing Silicone Tubes to the Nasal Cavity

Initially, the probes are passed through the lacrimal sac flap. Subsequently, two metallic parts are cut away and the two ends are tied together. Since this step may occur before the creation of the nasal flap, caution must be exercised to keep the tubes away from the knife using retractors to prevent unintentional cutting. The distal tip of the tie is then grasped using curved mosquito forceps and passed parallel to the nasal ridge within the middle meatus (Figure 2H). The silicone tube is then forcefully drawn and tied together multiple times under tension. The distal redundant part is cut, and upon release, the silicone tube coils back into the nose (Video 1). Silicone tubes can typically be removed after at least 4 weeks following the procedure.

**Surgical Gem:** Avoid overly tight tying of the silicone tube, as it may lead to cheese wiring of the punctum, which could result in dysfunction of the drainage system despite anatomical patency.

Step 7: Suturing the Flaps and Orbicularis Oculi Muscle

Suturing the flaps

Suturing the flaps, along with the orbicularis oculi muscle, in a layered fashion using Vicryl 6-0 (Figure 2I) is essential. In cases of overriding, it's preferable to prioritize nasal mucosal overriding.

**Surgical Gem: **If flaps are unavailable due to tissue loss, satisfactory results may still be achieved by suturing the overlying orbicular muscle alone.

Step 8: Suturing the Skin

Skin closure is performed using a continuous subcuticular technique with a nylon 6-0 suture (Figure 2J). Sutures can typically be removed 5-7 days post-operation.

Measures to reduce hemorrhage in external DCR surgery

In external DCR surgery, minimizing hemorrhage is crucial for successful outcomes. During DCR, sources of bleeding may include the angular vein, soft tissue, lacrimal sac, periosteum, nutrient-perforating arteries of the bone, or nasal mucosa. Various measures are employed to achieve this goal. Preoperative assessments are conducted to rule out bleeding disorders and assess blood pressure. During surgery, local anesthesia containing adrenaline is administered at the incision site to reduce bleeding. Maintaining low blood pressure and positioning the patient in a reverse Trendelenburg position helps minimize intraoperative bleeding. Additionally, nasal packing soaked in adrenaline is inserted before surgery to enhance hemostasis. Cautery is used judiciously to control bleeding, and known blood vessels are carefully avoided to prevent inadvertent bleeding. Effective suction is employed to remove blood and fluids from the surgical site, and the wound is packed with adrenaline-moistened gauze and surgical cellulose sponge to aid in hemostasis [9]. These measures collectively contribute to reducing hemorrhage and ensuring a successful external DCR procedure (Table 1).

**Table 1:** Measures to reduce hemorrhage in external DCR surgery DCR: Dacryocystorhinostomy

Measure	Description
Preoperative assessment	Comprehensive assessment to rule out bleeding diathesis and assess blood pressure.
Local anesthesia with adrenaline	Infiltration of local anesthesia mixed with adrenaline (1:100,000 to 1:200,000) at the incision site
Maintain low blood pressure	Efforts to keep blood pressure low during surgery to reduce the risk of bleeding.
Reverse Trendelenburg position	Positioning the patient with the head elevated to minimize bleeding.
Nasal packing with adrenaline	Insertion of ribbon gauze soaked in adrenaline into the middle meatus.
Judicious use of cautery	Careful and selective use of cautery to control bleeding during the procedure.
Avoidance of known blood vessels	Identification and avoidance of known blood vessels to prevent inadvertent bleeding.
Well-powered suction	Utilization of effective suction to remove blood and fluids from the surgical site.
Packing wound	Packing the wound with adrenaline/saline-moistened gauze and nasal packing aid in hemostasis.
Utilization of assisted bone removal techniques	Drill-assisted or ultrasonic bone aspiration osteotomy may reduce bleeding.

## Discussion

The success of external dacryocystorhinostomy (DCR) is closely linked to attaining a patent and appropriately sized ostium, coupled with minimal scarring. This can be accomplished through the judicious removal of bone, delicate handling of soft tissues, and careful management of flaps, potentially resulting in enhanced clinical outcomes by minimizing mucosal injury. Intraoperative bleeding can compromise visualization, thereby potentially impacting final surgical outcomes. Assisted osteotomy techniques, such as drill-assisted, offer the advantage of thermal cauterization of perforating bone vessels, potentially reducing intraoperative bleeding. The emergence of drill-assisted DCR methods aims to enhance procedural precision in judicious osteotomy while mitigating the risks associated with soft tissue and flap trauma, ultimately facilitating the creation of a more expansive patent ostium. However, a significant gap exists in the literature concerning comprehensive studies comparing traditional external DCR with drill-assisted techniques [2-5].

While drills have been extensively employed in neurosurgery, otolaryngology (ENT), and orthopedics for an extended period, their application in ophthalmology remains relatively underexplored. Despite their proven efficacy and safety in other surgical disciplines, the utilization of drills in ophthalmic procedures, particularly in DCR, has not received widespread investigation. This lack of exploration underscores a potential gap in leveraging established surgical tools and techniques from other specialties to enhance precision and outcomes in ophthalmic surgeries, highlighting the need for further research and evaluation in this domain.

Drill-assisted DCR is a standard procedure in the ophthalmology department where the procedure was filmed. The ophthalmologists shared an operation hall with the otolaryngology (ENT) department, providing valuable access to necessary equipment like drills. Over time, exchanging experiences with our ENT colleagues has facilitated the seamless integration of this technique into our routine practice.

A recent study comparing outcomes in external DCR using a piezoelectric ultrasonic bone aspirator (UBA) to a high-speed electric drill with a diamond burr technique demonstrated comparable outcomes and complication rates. Our experience with drill-assisted DCR aligns with these findings, as we have observed a reduction in the risk of flap injuries and hemorrhage during surgery, along with minimal complications and shorter surgical time. Utilizing assisted external DCR techniques represents a significant advancement in surgical precision, addressing longstanding challenges associated with the procedure [3,7].

A comparative analysis presented in Table 2 outlines various aspects of drill-assisted DCR and conventional external DCR. Drill-assisted DCR generally shows advantages in terms of shorter surgical duration, lower intraoperative hemorrhage rate, more regular osteotomy edge, increased anatomical patency, and potential prevention of soft tissue or mucosal injuries. However, it demands enhanced hand and foot coordination. In contrast, conventional DCR may face challenges such as longer surgical duration, higher bleeding rates, less regular osteotomy edges, and a risk of Sump syndrome. Additionally, it may require greater force in cases of a thicker frontal process of the maxilla [3,7].

**Table 2:** Comparison of drill-assisted and conventional external DCR DCR: Dacryocystorhinostomy Sump syndrome manifests when a residual nasolacrimal sac persists, accumulates fluids, and results in excessive tearing.

Aspects	Drill-assisted external DCR	Conventional external DCR
Surgical duration	Shorter	Longer
Intraoperative hemorrhage rate	Lower	Higher
Ostium edge	More regular and precise	Less regular
Anatomical patency	More patency	Possible Sump syndrome
Soft tissue and mucosal injury	Possible thermal injury and wrap-around shaft	Punch-out and mechanical injuries
Surgeon requirements	Hand and foot coordination	A greater force with a thicker frontal process of the maxilla

Despite the evident benefits of drill-assisted external DCR, there remains a scarcity of comprehensive reports in the literature. The limited number of studies impedes a thorough understanding and appreciation of the technique's nuances [3-7]. Moreover, there is a notable absence of comparative analyses, particularly in direct comparison with the conventional method. Comparative data on factors such as osteotomy edge regularity, ostium patency, soft tissue, and mucosal injuries, as well as surgeon requirements, would be particularly valuable for the surgical community.

Utilizing drill-assisted external DCR represents a significant advancement in precision within the surgical domain. The intricate nature of the procedure, characterized by a confined surgical field and the simultaneous use of multiple instruments, has traditionally posed challenges for documentation. Procedure filming is inherently difficult, further complicated by potential hemorrhages. However, the current video report successfully addresses these challenges, contributing valuable visual insights to the limited existing literature [3].

While this report offers valuable insights into the drill-assisted external DCR technique, several limitations must be acknowledged. Firstly, the study is based on a single-center experience, potentially limiting the generalizability of the findings to other institutions and patient populations. Additionally, the lack of a specified sample size and long-term follow-up data on patient outcomes hinder a comprehensive assessment of the technique's effectiveness and safety. Furthermore, while the video component enhances the visualization of the procedure, it may not fully convey the complexities or address potential intraoperative challenges. Moreover, the report does not discuss potential drawbacks of the drill-assisted approach, such as increased cost or the learning curve associated with adopting new surgical techniques. Acknowledging these limitations is essential for understanding the full scope and implications of implementing drill-assisted DCR in clinical practice.

Continued research efforts, including comparative studies and comprehensive outcome assessments, are warranted to further elucidate the potential benefits and limitations of drill-assisted DCR in clinical practice.

## Conclusions

In conclusion, this technical report provides a detailed visual guide to drill-assisted external dacryocystorhinostomy (DCR) with silicone intubation, addressing key surgical considerations and challenges. The report aims to optimize outcomes and minimize complications in drill-assisted DCR procedures by highlighting crucial steps and emphasizing meticulous technique. The significance of achieving a patent ostium with minimal scarring and the potential impact of intraoperative bleeding on final surgical outcomes is underscored. Assisted osteotomy techniques offer promising advantages in reducing bleeding and enhancing precision, contributing to improved surgical outcomes. Despite the evident benefits, further research is needed to comprehensively evaluate the comparative effectiveness and safety of drill-assisted DCR compared to conventional methods. Through continued exploration and dissemination of knowledge, integrating drill-assisted techniques into routine practice holds promise for advancing the surgical precision of external DCR.
